# Reaching further by Village Health Collaborators: The informal health taskforce of Vietnam for COVID-19 responses

**DOI:** 10.7189/jogh.10.010354

**Published:** 2020-06

**Authors:** Bach Xuan Tran, Hai Thanh Phan, Thao Phuong Thi Nguyen, Men Thi Hoang, Giang Thu Vu, Huong Thi Lei, Carl A Latkin, Cyrus SH Ho, Roger CM Ho

**Affiliations:** 1 Institute for Preventive Medicine and Public Health, Hanoi Medical University, Hanoi, Vietnam; 2 Bloomberg School of Public Health, Johns Hopkins University, Baltimore, Maryland, USA; 3 Institute for Global Health Innovations, Duy Tan University, Da Nang, Viet Nam; 4 Faculty of Medicine, Duy Tan University, Da Nang, Vietnam; 5 Center of Excellence in Evidence-based Medicine, Nguyen Tat Thanh University, Ho Chi Minh City, Vietnam; 6 Department of Psychological Medicine, National University Hospital, Singapore, Singapore; 7 Department of Psychological Medicine, Yong Loo Lin School of Medicine, National University of Singapore, Singapore, Singapore; 8 Institute for Health Innovation and Technology (iHealthtech), National University of Singapore, Singapore, Singapore

Since the first COVID-19 case importation on January 23, Vietnam is now the only country in the world contain the epidemic with 268 confirmed cases (52 active and 216 recovered) with no deaths over the past three months. Being proactive in contact tracing and restrict quarantine of multiple clusters early are common policies that helped Vietnam efficiently control the spread of COVID-19. However, they are resource-intensive while the country has a limited capacity for massive testing. How to ensure surveillance and detection at the grassroots level, especially in rural and remote areas, is a critical question that is challenging not only Vietnam but also other low- and middle- income countries. In this viewpoint, we first summarize the roles and responsibilities of the network of village health workers over its long history of development in Vietnam. Thereafter, we discuss the functionalities of this informal taskforce in monitoring COVID-19 epidemic and delivering packages of interventions, especially in disadvantaged settings.

Sharing a borderline of nearly 1400 km with China, it has been speculated that the northern mountainous region of Vietnam is at substantial risk of COVID-19 transmission [1]. In addition to the high cross-border trade volume, the Vietnam-China borderline is uniquely characterized by a mixed residence as well as farming in border regions through generations. It is a commonplace that border dwellers cross the border to visit their ancestral relatives or earn a living on a regular basis [1,2]. The region is also the fatherland of various ethnic minorities with distinct traditions and lifestyles. While the cultural diversity makes a great contribution to the beauty of the nation, differences in beliefs, languages and daily practices among the ethnic groups have put up barriers for the effectiveness of health education programs and preventive interventions. Scarcity of communication facilities, such as cable television and internet connection, also enhances the vulnerability of this population in the COVID-19 pandemic, in which health education and communication programs have proved its strength in preventing the transmission of the virus. Therefore, it is critical to effectively and efficiently mobilize the available resources to address the current obstacles.

**Figure 1:**
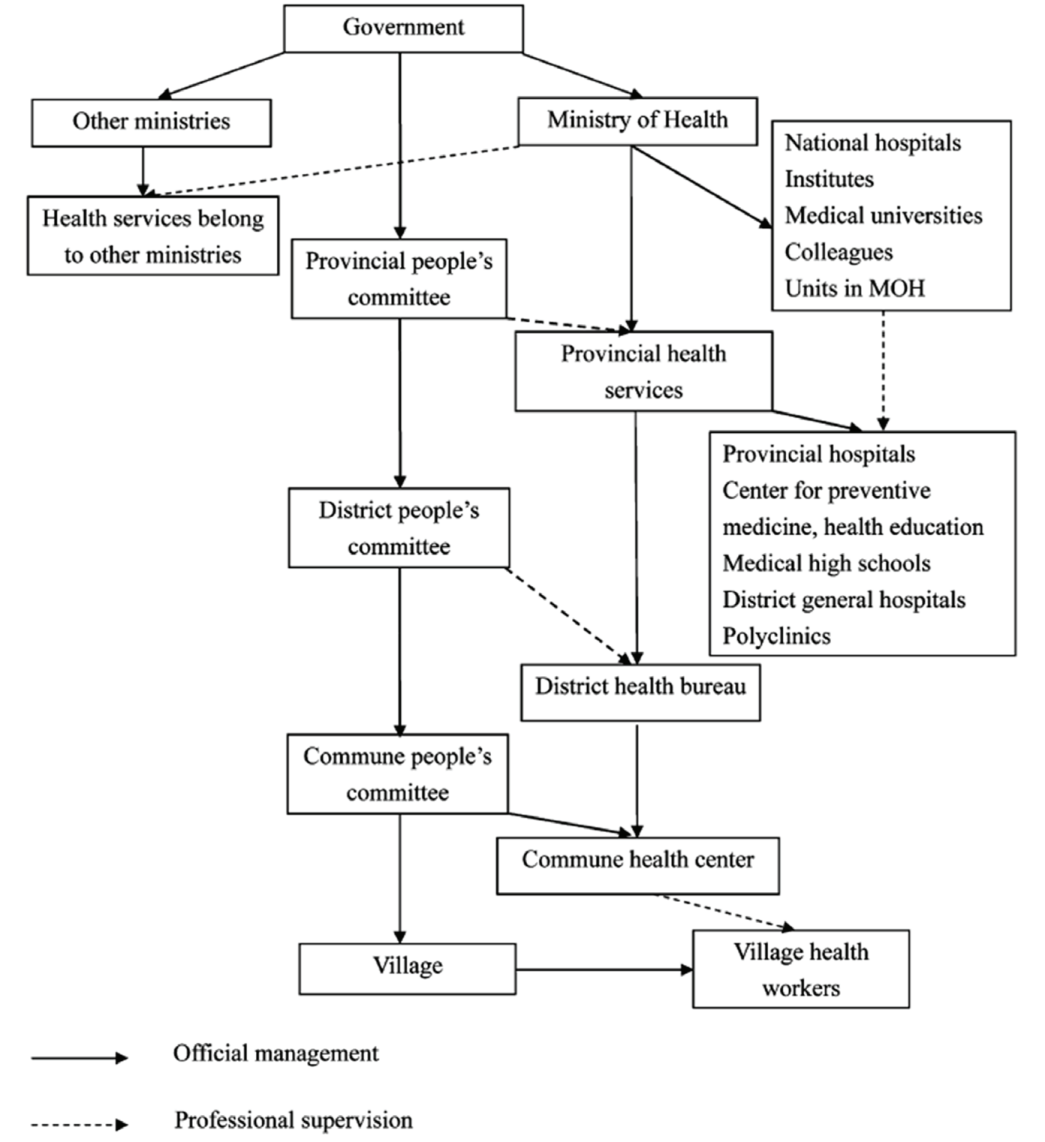
Outline of the Vietnamese health system. (Source: Le D-C et al., 2010[3]).

In Vietnam, village health care is part of the grassroots health system and playing an important role in primary health care (**Figure 1**) [3]. In the mountainous areas, village health workers usually live in the villages and hamlets with the residents, and thus, they are more than health professionals but neighbors and friends. Village health workers thus have an understanding about people’s life, financial situation and health status of each family, as well as the accessibility and affordability of available local health services. Understanding the need of the residents and being aware of the importance of village health care in promoting public health, village health workers are capable of making substantial contributions to not only the implementation of programs but also the development of fruitful intervention focus [4].

**Figure:**
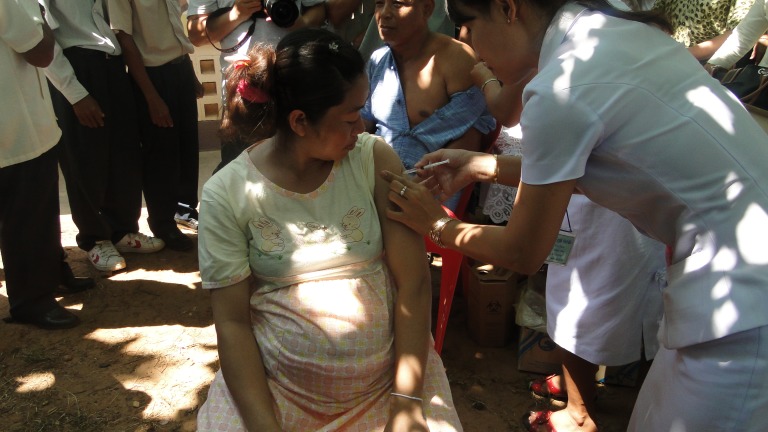
Photo: A pregnant woman vaccinated with the flu vaccine in Vietnam. (Source: CDC Global, available at https://flic.kr/p/kqFmRi).

Understanding the important role of village health care in primary health care in the community, Vietnamese government and Ministry of Health issued a number of decisions on strengthening and empowering the grassroots health care network, including village health services. The Health Minister's Decision No. 3653 on November 15, 1999 of the Health’s Minister defined the functions and tasks of village health workers, which provided village health workers with clear guides on their functions and duties in the care and protection of people's health. The decision introduced five major responsibilities of village health workers, namely health education and communication, instruction of performing hygiene for disease prevention, maternal and child health care and family planning, first aid and basic medical care, and implementation of health programs [5]. In addition, a series of legal documents were amended and replaced to clarify the functions and duties of village health workers [6,7]. According to the regulations of the Ministry of Health in Circular No. 07/2013/TT-BYT on standards, other functions and responsibilities of village health services are: implementing health education and communication program in the community (propagandizing and disseminating knowledge about health protection, environmental sanitation and food safety, guiding a number of initial health care measures); disease prevention in the community; propagating and educating people about HIV/AIDS prevention and control; mobilizing, providing information and consulting on population and family planning work [8]. Nevertheless, village health care activities have been facing various difficulties, and complications since their main beneficiaries live in mountainous and rural areas of the country with many different occupations and variety of lifestyles and contexts as mentioned above [4].

COVID-19, which is caused by the new strain of coronavirus SARS-CoV-2, is an infectious respiratory disease that can result in pneumonia in humans [9]. Since the first case of COVID-19 was identified in China in December 2019, there have been a total of nearly 2.5 million of confirmed cases, with the death toll of more than 170 000 over 213 countries and territories [9]. The virus can be spread by human-to-human transmission, through droplets produced by infected people. When a person coughs or exhales, the droplets containing SARS-CoV-2 land on objects and surfaces around the person and other people can be infected as they contact those surfaces and touch their eyes, nose or mouth. People can also catch COVID-19 by breathing in droplets from a person with COVID-19 [10]. Infected people with no or mild symptoms are also potential sources with a high risk of transmission since they tend to not take the symptoms seriously and thus, ignore the necessary preventive measures [10]. In rural and mountainous areas, the risk of transmission is even higher due to the low coverage of health education and communication programs, limit in financial and human resources as well as existing cultural customs and health care beliefs. While in the urban areas and large provinces and cities, the information about appropriate preventive practices towards COVID-19 is distributed to every individual on a daily basis, in the disadvantaged areas, the exposure of people to such important knowledge remains relatively limited due to the lack of stable cable television and internet connection. In addition, there is a scarce of personal protective equipment and hygiene supply, such as facemask and hand sanitizer for people, disposable costumes and eye gear for health care workers. On the other hand, many members of the ethnic groups still prefer to seek the help of a religious intermediary or a fold healer instead of following the guidelines of health care workers and receiving formal medical care, which in turn heightens the risk of transmission.

In the fight against COVID-19, it is essential to mobilize existing resources and provide materials and skills as well as lean management for at-risk communities to efficiently detect early signs of localized transmissions, identify infected areas and effectively quarantine them. In order to do so, Village Health Collaborators should be quickly equipped with knowledge and skills in COVID-19 control and prevention through multilingual training materials delivered by both local and national trainers. Governors should set up mechanisms and protocols for information exchange, data synthesis, sample collection, and surveillance. It is also important to notice that some important skills are missing, including the identification of mental health symptoms, mapping most vulnerable groups, such as the elderly, the poor, etc. and community-based rapid risk assessment. Counselling and social support should also be incorporated as parts of comprehensive approaches for those living in disadvantaged areas.
